# Comparison of Teratoma Formation between Embryonic Stem Cells and Parthenogenetic Embryonic Stem Cells by Molecular Imaging

**DOI:** 10.1155/2018/7906531

**Published:** 2018-03-25

**Authors:** Hongyan Tao, Xiaoniao Chen, Anbang Wei, Xianghe Song, Weiqiang Wang, Lu Liang, Qinjun Zhao, Zhibo Han, Zhongchao Han, Xiaojing Wang, Zongjin Li

**Affiliations:** ^1^Nankai University School of Medicine, Tianjin 300071, China; ^2^The Key Lab of Bioactive Materials, Ministry of Education, The College of Life Science, Nankai University, Tianjin 300071, China; ^3^Department of Ophthalmology, Ophthalmology and Visual Science Key Lab of PLA, Chinese PLA General Hospital, Beijing 100853, China; ^4^State Key Laboratory of Kidney Diseases Chinese PLA General Hospital, Beijing 100853, China; ^5^Faculty of Clinical Medicine, Zhejiang University School of Medicine, Zhejiang 310058, China; ^6^Department of Cardiology, Rizhao Hospital of Traditional Chinese Medicine, Shandong 276800, China; ^7^Beijing Engineering Laboratory of Perinatal Stem Cells, Beijing Institute of Health and Stem Cells, Health & Biotech Co., Beijing 100176, China; ^8^State Key Lab of Experimental Hematology, Chinese Academy of Medical Sciences & Peking Union Medical College, Tianjin 300020, China

## Abstract

With their properties of self-renewal and differentiation, embryonic stem (ES) cells hold great promises for regenerative therapy. However, teratoma formation and ethical concerns of ES cells may restrict their potential clinical applications. Currently, parthenogenetic embryonic stem (pES) cells have attracted the interest of researchers for its self-renewing and pluripotent differentiation while eliciting less ethic concerns. In this study, we established a model with ES and pES cells both stably transfected with a double-fusion reporter gene containing renilla luciferase (Rluc) and red fluorescent protein (RFP) to analyze the mechanisms of teratoma formation. Transgenic *Vegfr2-luc* mouse, which expresses firefly luciferase (Fluc) under the promoter of vascular endothelial growth factor receptor 2 (*Vegfr2-luc*), was used to trace the growth of new blood vessel recruited by transplanted cells. Bioluminescence imaging (BLI) of Rluc/Fluc provides an effective tool in estimating the growth and angiogenesis of teratoma *in vivo.* We found that the tumorigenesis and angiogenesis capacity of ES cells were higher than those of pES cells, in which VEGF/VEGFR2 signal pathway plays an important role. In conclusion, pES cells have the decreased potential of teratoma formation but meanwhile have similar differentiating capacity compared with ES cells. These data demonstrate that pES cells provide an alternative source for ES cells with the risk reduction of teratoma formation and without ethical controversy.

## 1. Introduction

ES cells are unique among all stem cell populations owing to their high pluripotency and differentiation capacity, which makes them one of the most promising cells for regenerative medicine [1, 2]. Currently, successful differentiation methods of ES cells have been developed into multiple tissue types, including bladder [3], pancreas [4], liver [5], and female reproductive [6]. However, the possibility of teratoma formation after cell transplantation has restricted their applications in clinical [7]. More importantly, ethical concerns limit the isolation and application of human ES cell in clinical translation. Recently, parthenogenetic embryonic stem (pES) cells have attracted the interest of researchers for their pluripotent differentiation without ethical issues [8]. These cells can be derived from embryos resulted from artificial activation of oocytes without fertilization [9, 10]. The pES cell lines are similar to ES cells in terms of proliferation, expression of pluripotency markers, and capacity to differentiate into several cell lines including tenocyte-like cells [11], osteogenic cells [12], and neural cells [12].

Although the biological characterization of pES cells is well documented, available analysis about the biological behavior and teratoma formation mechanism of pES cells is limited. Thus, a detailed observation and functional analyses between pES cells and ES cells would gain insight into the teratoma formation of cells from different sources. To date, despite several attempts at blocking teratoma formation, including introduction of suicide genes [13], inhibition of cell-cycle regulatory proteins [14], immunodepletion [15], selecting the desired cell type [16], or introducing cytotoxic antibody [17], a clinically viable strategy to eliminate teratoma formation needs to be developed [18]. In previous study, after establishment *in vivo*, the typical behavior of teratoma is remodeling microenvironment especially the formation of blood vessels for nutrients to support their growth [19], in which angiogenesis plays a crucial role. Therefore, monitoring the processes of teratoma angiogenesis may provide novel approach for inhibiting the formation of teratoma and direct future clinical translational application of pES cells.

In this study, we developed a teratoma model to monitor the behavior of pES and ES cells in transgenic mice by molecular imaging. A lentiviral vector carrying EF1*α* promoter, which drives double-fusion construct containing renilla luciferase (Rluc) and red fluorescent protein (RFP) reporter genes, was used to achieve localization of the transplanted cells [20, 21]. Molecular imaging provides the possibility to visually monitor the cellular processes after transplantation, including proliferation and angiogenesis. In addition, transgenic *Vegfr2-luc* mice expressing Fluc under the promoter of *Vegfr2-luc* allow us to capture and quantify teratoma angiogenesis *in vivo.*

## 2. Materials and Methods

### 2.1. Cell Culture

ES cell line was obtained from fertilized embryos of mice while pES cell line was isolated from activated oocytes [22]. These two cell lines were maintained with DMEM (Gibco, Grand Island, NY) on mouse embryonic fibroblast (MEF) feeder layers, which were preliminary treated with 10 *μ*g/ml mitomycin C. The medium contains 15% ES-qualified fetal bovine serum (FBS; HyClone, Australia), 0.1 mM *β*-mercaptoethanol, 1% nonessential amino acids (NEAA; Gibco), 1% penicillin/streptomycin (Gibco), and 1000 units/ml leukemia inhibitory factor (LIF; Millipore). To detect the trace of transplanted cells *in vivo*, pES cells and ES cells were transduced with lentiviral vector, which carries an EF1*α* promoter, driving renilla luciferase (Rluc) and red fluorescent protein (RFP) double-fusion reporter genes (RR), and were named pES-RR and ES-RR, respectively. A bright micrograph of each group was taken to observe cells' morphology. Culture medium was changed daily, and pES-RR or ES-RR was passaged once every two days.

### 2.2. Characterization of Reporter Gene-Labeled Cells

The expression of RFP in reporter gene-labeled cells was observed with an inverted fluorescence microscope; meanwhile, the activity of Rluc in these cells was measured by bioluminescence imaging (BLI). BLI was performed using IVIS Lumina II system (Xenogen Corporation, Hopkinton, MA) as described [23]. In sequential noninvasive imaging, pES-RR or ES-RR were cultured in a 24-well plate and then exposed to 1 *μ*g/ml of coelenterazine (NanoLight, Technology, Pinetop, AZ) directly. Imaging was performed immediately with a cooled charge-coupled device (CCD) bioluminescence camera for 3 min. Subsequently, bioluminescence was quantified in units of maximum photons per second per centimeter square per steradian (p/s/cm^2^/sr). A linear relation between signal intensity and cell number was analyzed using Graphpad Prism 6.0 Software (GraphPad Software Inc., San Diego, CA).

### 2.3. Cell Proliferation Assay

Cell proliferation was determined using a trypan blue (Gibco) exclusion assay in triplicate, and population doublings were calculated. pES cells, ES cells, and reporter gene-labeled cells were seeded in 24-well plate at a density of 2 × 10^4^ per well. Five fields per well were selected randomly and marked carefully. Cells in the chosen fields were counted at 24, 48, and 72 h time points.

### 2.4. Alkaline Phosphatase Staining

To determine the undifferentiated state of transduced cells, alkaline phosphatase (ALP) activity was measured using alkaline phosphatase kit (Sigma-Aldrich, St. Louis, MO). ES cell, pES cell, and reporter gene-labeled cells were first fixed in 10% formalin, then cells were washed with deionized water for 30 s and incubated with alizarin red premixed solution in a dark environment for 15 mins at room temperature. After a brief rinse, samples were counterstained for 30 s and then washed thrice to remove the dissociative dye. After drying, staining cells were observed with an optical microscopy [24].

### 2.5. Embryoid Body Formation

To detect differentiation capacity of pES-RR and ES-RR, cells were trypsinized to carry out a single-cell suspension and achieved at a density of 1 × 10^5^ per ml in LIF-deficient ES cell medium. Then, 20 *μ*l cell suspension was seeded on uncoated Petri dishes using hanging drop culture [25]. Primary embryoid body (EB) was formed after 48 hours and then replanted into a 6-well plate-coated gelatin previously cultured with 2 ml ES cell medium without LIF. EBs of each group were harvested at day 6 and day 12 for following gene expression analysis.

### 2.6. Real-Time Polymerase Chain Reaction

Teratoma from day 28 was analyzed by quantitative real-time polymerase chain reaction (qPCR). Total RNA was extracted from the samples with TRIzol reagent (Invitrogen, Grand Island, NY) according to instructions supplied by the manufacturer. Then, 2 *μ*g of total RNA was used for the first-strand cDNA template synthesis, and the manipulation was the same as above. Afterwards, qPCR was performed in triplicate on Opticon® System (Bio-Rad, Hercules, CA) and the relative gene expression level of vascular endothelial growth factor A (VEGFA), VEGFR2, CD34, angiopoietin-1 (Ang-1), angiopoietin-2 (Ang-2), and tyrosine kinase with immunoglobulin-like loops and epidermal growth factor homology domains 2 (Tie-2) was quantified using TransStart Top Green qPCR SuperMix Kit (TransGen Biotech, China). Relative mRNA folding changes were determined by the 2^−ΔΔCT^ method.

### 2.7. Teratoma Model

The experiments were approved by the Nankai University Animal Care and Use Committee. All protocols were performed in strict accordance with the Guidelines for the Care and Use of Laboratory Animals published by the US National Institutes of Health (8th edition, 2011). 8–10-week-old female transgenic mice (Xenogen Corp., Hopkinto, MA), expressing firefly luciferase (Fluc) under the promoter of murine vascular endothelial growth factor receptor 2 (*vegfr2*), were housed under standard laboratory conditions [26]. Mice were injected with 3 × 10^6^ of pES-RR into the right fourth pair of mammary fat pad and 3 × 10^6^ of ES-RR into the opposite site in 100 *μ*l of phosphate-buffered saline (day 0). Teratoma development was tracked by BLI of Rluc, and tumor angiogenesis was evaluated by BLI of Fluc simultaneously. All mice were euthanized and teratomas were harvested for further analysis 4 weeks posttransplantation (day 28).

### 2.8. Semiquantitative RT-PCR

Complementary DNA (cDNA) template was synthesized from 2 *μ*g of total RNA isolated from EBs collected in advance by using TIANScript RT kit (TIANGEN Biotech, China) and diluted to 50 *μ*l. 1 *μ*l of first-strand cDNA mixture was added to the reverse transcriptase polymerase chain reaction (RT-PCR) system. Ectodermal-, mesodermal-, endodermal-, and pluripotent-specific marker (Nestin, Brachyury, Gata6, Oct4 and Nanog) were compared among all groups.

### 2.9. Optical Bioluminescence Imaging

Imaging of Rluc and Fluc expression was aimed at monitoring teratoma development and tumor angiogenesis, respectively. Tumor BLI was performed using IVIS Lumina II system (Xenogen Corporation, Hopkinton, MA) [27]. In short, 2.5 mg/kg of coelenterazine was injected intravenously into transgenic mice, and mice were imaged for 1-2 min to assess Rluc expression immediately. In addition, the same mice with intraperitoneal injection of 150 mg/kg of reporter probe D-Luciferin (Biosynth International, Naperville, IL), were imaged for 1–10 min to evaluate Fluc expression. All animals were scanned from day 0 to day 28. Region of interest (ROI) was drawn over the signals on both mammary fat pads, and imaging signals were analyzed as previously mentioned [28].

### 2.10. Histological Analysis

The isolated teratoma was fixed in 4% paraformaldehyde and dehydrated in 30% sucrose overnight. Subsequently, these samples were embedded into optimal cutting temperature (OCT) compound (Sakura Finetek, Japan) or paraffin, respectively. Frozen samples or paraffin specimens were both cut into 5 *μ*m thick sections for next staining. For immunofluorescence staining, rat anti-mouse CD31 antibody (BD, San Jose, CA) and Alexa Fluor 488 fluorescent secondary antibody were used to determine angiogenesis in teratomas. Nuclei were visualized with 4,6-diamidino-2-phenylindole (DAPI; Southern Biotech, Birmingham, AL), and four representative fields were measured to detect microvascular density (MVD) under fluorescence microscope. Moreover, to investigate differentiation status of teratomas, five consecutive sections of each paraffin sample were stained with hematoxylin and eosin (HE).

### 2.11. Statistical Analysis

Statistical analysis was accomplished by using Graphpad Prism 6.0 Software (Graphpad Software Inc.). Two-way repeated measures ANOVA as well as two-tailed Student's *t*-test were used. Differences were considered significant at *P* values of <0.05. Unless specified, data were given as mean ± SEM.

## 3. Result

### 3.1. Labeling of pES Cells and ES Cells with DF Reporter Genes

**Figure 1:**
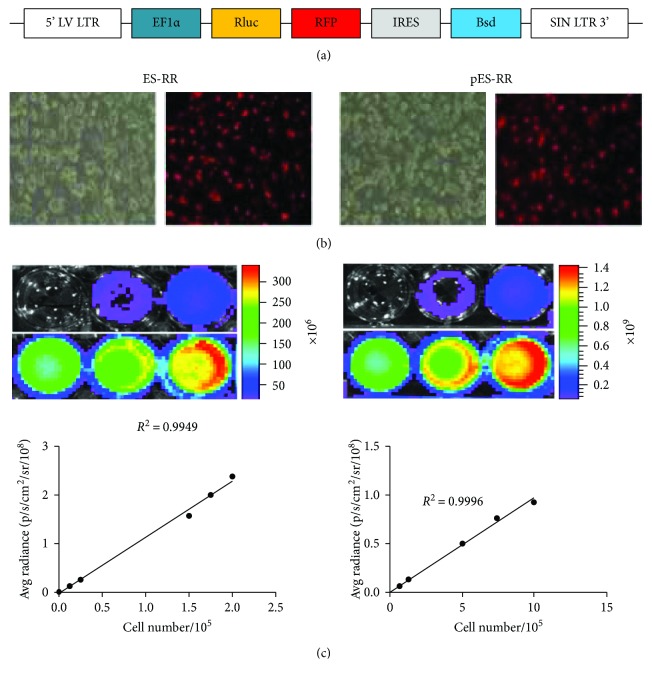
Generation of pES cells and ES cells labeled with double-fusion reporter genes. (a) Schema of lentiviral construct showing EF1*α* promoter driving Rluc and RFP. (b) Brightfield and fluorescence microscopy showing RFP expression in pES cells and ES cells. (c) BLI of pES cells and ES cells shows a robust correlation between cell number and Rluc activity.

To monitor the dynamic processes in teratoma development, we created two cell lines, pES-RR and ES-RR, labeled with double-fusion reporter genes (Figures 1(a) and 1(b)). Positive RFP cells were screened by Bsd (Blasticidin), and immunofluorescence assay revealed robust expression of RFP. A strong correlation between Rluc activity and cell number was observed in both pES-RR and ES-RR using Xenogen IVIS system (Figure 1(c)), which demonstrated the possibility to assess cell number *in vitro* and teratoma growth *in vivo* by analyzing Rluc signal intensity. Cell number of labeled of pES-RR and ES-RR correlated linearly with Rluc activity (*R*^2^ = 0.99).

### 3.2. Characteristics of pES-RR and ES-RR

**Figure 2:**
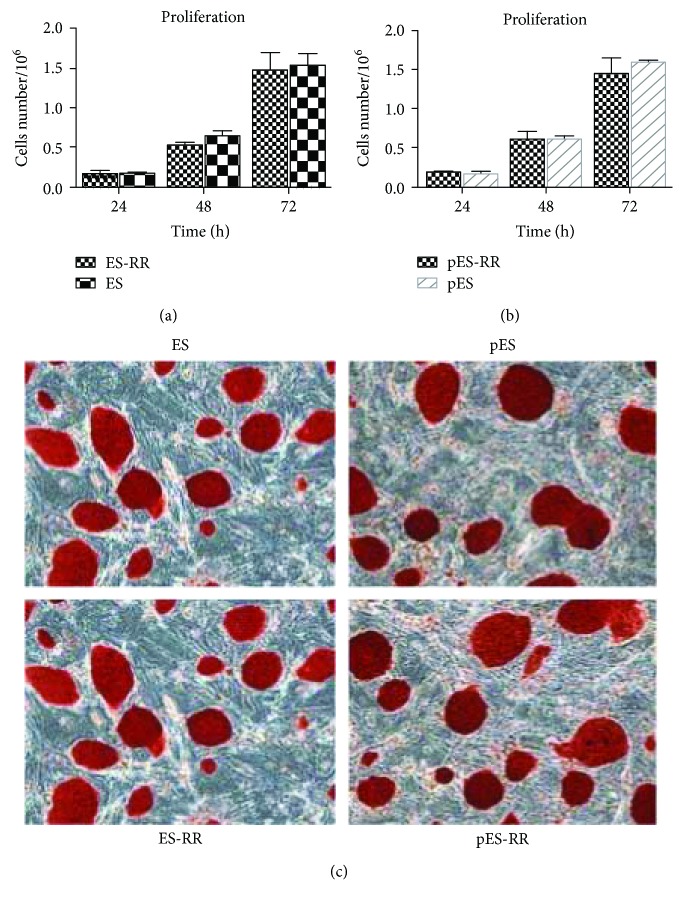
The characterization of reporter gene-labeled cells. (a, b) Cell proliferation activity between wild-type cells and labeled cells shows no significant difference *in vitro*. (c) The pluripotency of the cells was detected by ALP staining, and undifferentiated cells appear red, whereas MEF cells appear colorless. ALP staining of each group exhibited the transfected report genes does not affect the pluripotency of pES cells and ES cells.

After establishing these two cell lines, we examined the proliferation ability of ES-RR (Figure 2(a)) and pES-RR (Figure 2(b)) cultured in standard ES cell conditions. There is no significant difference between pES cells, ES cells, and their wild-type in live cell image and colony formation. These results proved that the transfection of reporter genes does not affect the proliferation ability of pES cells and ES cells. Simultaneously, according to the ALP staining on transfected cells (Figure 2(c)), there is no significant difference in pluripotency of transfected cells compared with wild-type cells. These data indicated that the transfection of reporter gene did not alter the characteristics of pES cells and ES cells, which means we could monitor the behavior of transplanted cells with the expression of reporter gene.

### 3.3. Visualization of the Teratoma Growth and VEGFR2 Expression Dynamic Processes *In Vivo*

**Figure 3:**
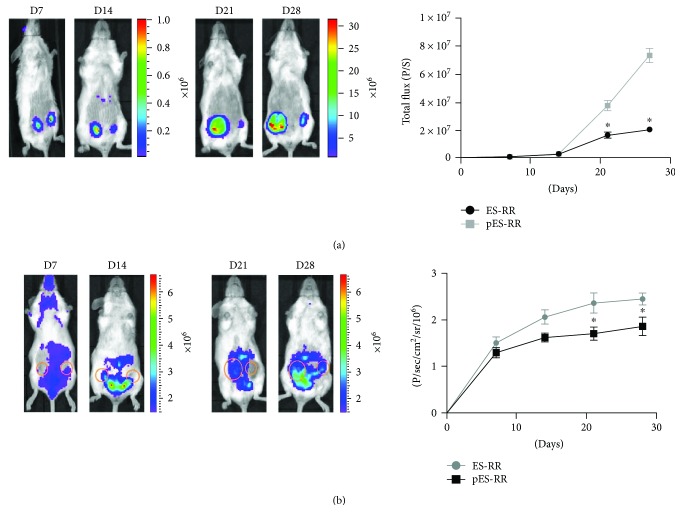
Visualization of the teratoma growth and VEGFR2 expression *in vivo*. (a) Rluc imaging of teratoma progression of Rluc signals showed that the teratoma formed in pES-RR group was smaller than that in ES-RR group. Quantification analysis demonstrated there was a significant difference between the two groups. (b) Fluc imaging and quantification analysis of VEGFR2 expression in transgenic mice revealed enhanced angiogenesis in ES cell-derived teratomas. ^∗^*P* < 0.05 compared with the ES-RR group.

The restriction of ES cells in potential clinical applications is mainly attributed to the teratoma formation. Therefore, a deeper research in the behavior of teratoma after cell injection is necessary and molecular imaging techniques were used to monitor this process. Within 4 weeks of transplantation, we performed BLI of Rluc in selected time points and analyzed with living imaging software. Results showed that pES-RR is similar to ES-RR. Both of them can form tumor *in vivo* at the 14th day after transplantation (Figure 3(a)), and the growth rate of tumor was increased over time. However, the tumor formed by pES-RR was significantly smaller than that of ES-RR. Such difference was constantly observed for the entire experimental period up to 28 days. Meanwhile, the formation and development of tumors require recruitment of new blood vessels, which plays an important role in microenvironment formation to support tumor growth. ES-RR and pES-RR were transplanted into *VEGFR2-luc* transgenic mice. The expression of VEGFR2 induced by angiogenesis could act as an indicator for blood vessel formation monitored by BLI in real time. Increased BLI signal was found in ES-RR group, which suggests that VEGF/VEGFR2 pathways were activated while ES cells have the enhanced capacity of recruiting for host blood vessels (Figure 3(b)).

### 3.4. Histological Analysis

**Figure 4:**
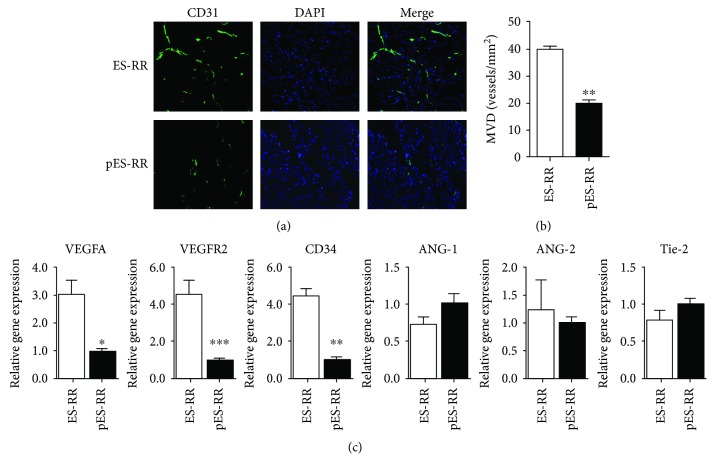
Analysis of teratoma angiogenesis. (a) Angiogenesis observed in pES-RR group and ES-RR group by CD31 immunofluorescence staining. (b) Microvessel density (MVD) of teratoma tissue was measured by computer image analyzing system. (c) The expression of CD34/VEGF/VEGFR2 was significantly lower in pES-RR group compared with ES-RR group, whereas there was no significant difference in the expression of ANG-1/ANG-2/Tie-2 between these two groups. ^∗^*P* < 0.05; ^∗∗^*P* < 0.01; ^∗∗∗^*P* < 0.001.

Molecular imaging revealed that teratoma formed by pES cells is significantly weaker than that of ES cells; we speculate on the two major causes of this difference, angiogenesis and multipotential differentiation. To determine the degree of angiogenesis after the cell transplantation, sections were stained with the anti-CD31 antibody. The expression of CD31 could reflect the angiogenesis in teratoma. Results showed that the density of new blood vessels of pES-RR group was significantly weaker than that of ES-RR group (Figures 4(a) and 4(b)).

This difference between pES-RR and ES-RR reminds us to gain insight into the mechanisms of blood vessel formation; we conducted a comparison of gene expression of these two groups. We detected the expression of angiogenesis-related genes including CD34, VEGFA, VEGFR2, Ang-1, Ang-2, and their receptor (Tie-2) by real-time PCR. The results showed that the expression of CD34/VEGF/VEGFR2 in pES-RR was significantly lower than that in ES-RR (*P* < 0.05 and *P* < 0.01). Meanwhile, there is no significant difference in the expression of Ang-1, Ang-2, and Tie-2 between pES-RR group and ES-RR group (Figure 4(c)). Those results indicate that pES-RR has weaker proangiogenic effect than ES-RR, and VEGF/VEGFR2 signal pathway plays a vital role in it.

### 3.5. Differentiation Potential of pES-RR and ES-RR

**Figure 5:**
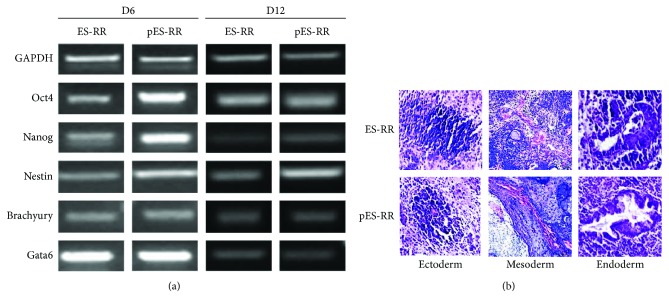
Differentiation potential between two cell lineages. (a) RT-PCR analysis of the expression of stemness-related genes of EBs from two groups in D6 and D12, which displayed no significant difference. (b) Teratoma formation of pES-RR and ES-RR. Histological analysis of teratoma identifying the presence of derivatives of all three germ layers: neural tissue (ectoderm), vascular tissue (mesoderm), and glandular tissue (endoderm).

After confirming the role of angiogenesis in teratoma formation, we want to test the differentiation potential of pES cells to make sure they retain an extensive differentiation capability *in vitro*. ES and pES cell differentiation were done by EB formation. EBs were collected after 48 hours and then cultured in differentiation medium to be spontaneously differentiated. The markers of three germ layers including gata6, brachy, nestin, and the stemness marker Oct4 on day 6 and day 12 were detected both in ES-RR group and in pES-RR group (Figure 5(a)). Results showed that there was no significant variation in the differentiation potential between pES-RR and ES-RR *in vitro*.

To further investigate the potential of the multidirectional differentiation between ES and pES cells, HE staining was applied for detection of the differentiation of three germ layers in the teratoma tissue. Through the staining results, we can find the markers of three germ layers in the teratoma tissue formed by both pES-RR and ES-RR: the ectoderm (nerve tissue), mesoderm (vascular system), and endoderm (glandular tissue) (Figure 5(b)). The gene expression shows that there is no obvious difference between these two cells in the ability of differentiation *in vitro* and *in vivo*.

## 4. Discussion

Recently, stem cell-based regenerative medicine is rapidly progressing as a promising tool for the repair and replacement of damaged cells, tissues, and organs. In this study, two kinds of stem cells, pES cells and ES cells, were obtained from hybrid mice to make a comparison of their teratoma-forming potentials. With transgenic *vegfr2-luc* mice [29], we observed transplanted stem cell behavior as well as angiogenesis process dynamically in living animals [21]. Results showed that pES is capable of differentiation into all three germ layers suggesting that there was no significant difference in differentiation potential between pES cells and ES cells. The VEGF/VEGFR2 pathway-related angiogenesis process was paralleled to teratoma growth proving that angiogenesis reflected the growth of teratoma and act as an important diagnosis and treatment target.

In the field of stem cell-based therapy, three types of cells, ES cells, somatic stem cells, and induced pluripotent stem cells (iPS cells), are mainly focused on. ES cells hold great potential in stem cell-based regenerative medicine for their capability to undergo unlimited self-renewal and differentiation. Compared with MSCs derived from mesodermal, ES cells are able to differentiate into every cell type [30], while the immunogenicity and safety of iPS cells produced from genetic method are considered major obstacles to regenerative medicine. ES cells with ability to differentiate into specific target tissues can eliminate some untreated diseases. For example, human ES cell-derived oligodendrocyte progenitors were used in the treatment of depletion of oligodendrocyte progenitors and demyelination following radiation [31].

However, many concerns are hindering the progress in ES cell-based replacement therapy, especially ethical issue, which cannot be solved by research or legislation. Therefore, some other cell resources are seeking to replace ES cells as agent of cell-based tissue regeneration. As a promising alternative, pES cells are further investigated. Their differentiation ability is considered to be similar with ES cells, while ethical issue is not existing in these cells. Recent studies have shown the treatment effects of pES cells in ischemic heart disease, neurodegenerative disease, and tendon injury indicate the active role of pES cells [11, 22, 32]. Although some researchers found that pES cells could form teratoma after cell transplantation, its occurrence and size are smaller than those in ES cells [22]. Thus, the elimination of the teratoma formation risk would greatly facilitate the development of stem cell replacement therapy using pES cells.

In summary, the teratoma model in our present study using transgenic mice with VEGFR2 promoter characterized the behavior of ES cells and pES cells after transplantation. BLI revealed survival and angiogenesis processes of these labeled cells after transplantation in real time. Overall, angiogenesis was paralleled to teratoma growth in initiation, development, and regression, which provides direct evidence for the role of angiogenesis in teratoma formation. Our study confirmed this effect and further demonstrated that VEGF/VEGFR2 signal pathway is closely related with angiogenesis during teratoma formation. We believe that an extensive understanding about teratoma formation mechanisms and angiogenesis stimulated by pES cells can help improve stem cell-based therapeutic in safety and efficacy.

## 5. Conclusion

Transplanted mouse pES cell-formed teratoma was significantly smaller than that produced by ES cells. These data revealed that the inhibition of VEGF/VEGFR2 signal pathway may reduce the probability of residual cells to form teratomas of ES and pES cells. Therefore, we believed that pES cells might provide safer sources for clinical translational application. However, detailed elucidation of the mechanisms of teratoma formation of ES and pES cells underlying the angiogenesis stimulation awaits further investigation.
